# Immunosuppressive mechanisms of human bone marrow derived mesenchymal stromal cells in BALB/c host graft versus host disease murine models

**DOI:** 10.1186/s40164-015-0007-0

**Published:** 2015-04-30

**Authors:** Joseph Delano Robles, Yin Ping Liu, Jiamin Cao, Zheng Xiang, Yin Cai, Michael Manio, Eva HC Tang, Godfrey Chi-Fung Chan

**Affiliations:** Department of Paediatrics and Adolescent Medicine, The University of Hong Kong Li Ka Shing Faculty of Medicine, Queen Mary Hospital, 102 Pokfulam Rd., HKSAR, PRC; Department of Pharmacology and Pharmacy, The University of Hong Kong Li Ka Shing Faculty of Medicine, Hong Kong, Hong Kong

**Keywords:** GVHD, MSC, Immunosuppression, Bone marrow transplantation

## Abstract

**Background:**

Mesenchymal stromal cells (MSCs) are proven to have immunosuppressive functions via various mechanisms. These mechanisms were demonstrated by administering bone marrow derived human MSCs (hMSCs) to graft versus host disease (GVHD) murine models.

**Methods:**

BALB/c host mice were irradiated prior to receiving C57BL/6 donor T cell depleted bone marrow (TCDBM) cells (negative control) and donor CD4+ T lymphocyte with (treatment group) or without hMSCs (positive control). The presence of hMSCs in target tissues and lymphoid organs was documented by using *in vivo* imaging and measuring the expression of EphB2 and ephrin-B2 by RTqPCR. Survival rate and GVHD score were also monitored. Tissue sections were obtained for histopathologic analysis. Flow cytometry was used to document donor T cell alloreactivity and expression of CCR5, CXCR3 and CCR7. ELISA was utilized to determine levels of proinflammatory cytokines, RANTES (CCL5) and phosphorylated STAT 5A/B. RTqPCR was performed to quantify expression of CCL3 and CXCL9. Western blotting was performed to qualitatively measure iNOS expression.

**Results:**

Survival rate and GVHD score improved with hMSC treatment. Pathologic changes of GVHD were abrogated. Documentation of suppression of RANTES, CCL3, CXCL9, CCR5 and CXCR3 with simultaneous decrease of donor T cell alloreactivity was demonstrated 6 days after transplantation, along with reduction of levels of inflammatory cytokines, suppression of STAT 5A/B phosphorylation, increased expression of CCR7 and increased production of nitrous oxide by hMSCs. Documentation of homing of hMSCs to lymphoid organs and target tissues was also performed.

**Conclusions:**

These mechanisms contribute to the current understanding of MSC mechanisms of immunosuppression and forms a comprehensive picture of how they exert immunosuppression in an *in vivo* model of immune dysregulation.

**Electronic supplementary material:**

The online version of this article (doi:10.1186/s40164-015-0007-0) contains supplementary material, which is available to authorized users.

## Background

Mesenchymal stromal cells (MSCs) were first identified from the bone marrow and are described as self-renewing cells capable of differentiating into cells of the mesodermal (bone, fat, cartilage cells) lineage [1]. *In vitro*, they are adherent to the surface of plastic plates and flasks. These cells do not express markers such as CD45, CD34, CD14 or CD11b, CD79a or CD19 and HLA class II [2]. Its characteristic multipotentiality makes it a promising candidate as an important agent in the field of cellular therapy. Studies that involve differentiation of MSCs into cells of the endodermal and ectodermal lineage are currently underway hence MSCs’ possible use in the fields of cardiovascular medicine, endocrinology, gastroenterology, neurology, orthopedics, pulmonology, and dermatology [3-9].

About a decade ago, bone marrow derived stromal cells were reported to be able to suppress T cell proliferation leading to various studies that attempted to explain the mechanisms of immunonodulation induced by MSCs and also paving the way for its probable clinical utility in graft versus host disease (GVHD) [10]. Most of these mechanisms were explained either by obtaining MSCs of murine sources and infusing them to mice with the alloreactive reaction dependent on the mouse immune system (i.e. non-humanized GVHD models) [11,12] or by acquiring hMSCs then administering them to immunodeficient mice whose immune system is reconstituted with human immune cells (i.e. humanized GVHD models) [13-15]. There are a few reports that used hMSCs administered to non-humanized GVHD model mice [16,17]. In this study we explained the immunosuppressive mechanisms of MSCs using bone marrow derived hMSCs infused to non-humanized GVHD mice.

## Results

### hMSCs migrate towards GVHD target organs

**Figure 1:**
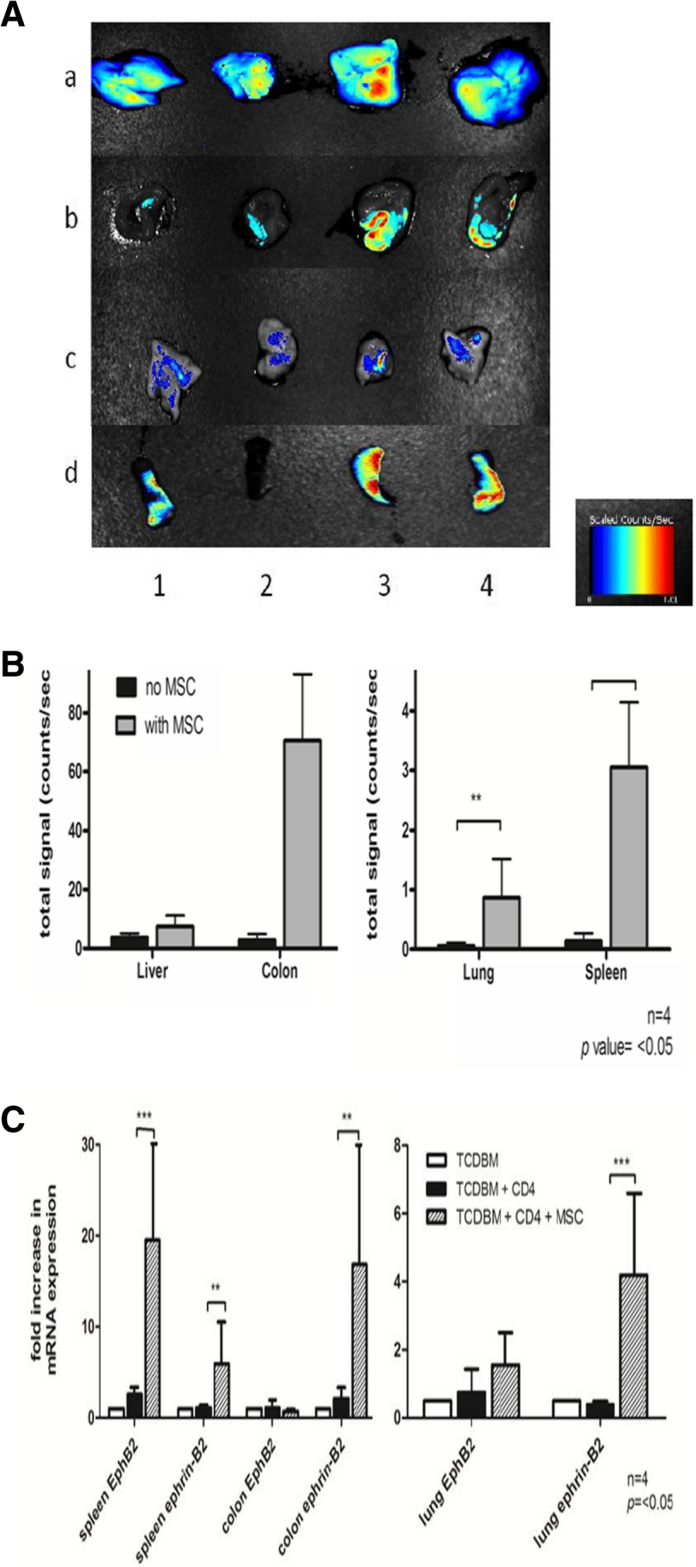
**Detection of hMSC homing capacity. (A-B)** *In vivo* imaging of hMSCs in GVHD host tissues. **(C)** mRNA levels of EphB2 and ephrin-B2 in GVHD host tissues. Lethally irradiated BALB/c host mice were given intravenous injections of 2 × 10^6. TCDBM cells and 0.25 × 10^6 CD4+ T cells from C57BL/6 donors with or without 1 × 10^6 hMSCs. hMSCs are stained with CM-Dil prior to injection to host mice. **(A)** Fluorescent signals from host liver (a), colon (b), lung (c)and spleen (d) 6d after transplantation without (1,2) or with (3,4) cotransplantation of hMSCs. Color bar represents signal intensity code. **(B)** Quantification of the signals in the liver, colon, lung and spleen. **(C)** Expression of EphB2 and ephrin-B2 6d after transplantation in BALB/c host spleen, colon and lung was measured by real-time RT-qPCR. Data were combined from 2 independent experiments with 2 mice in each experiment (n = 4 *p<0.05).

In order to identify the earliest time we would be able to detect the presence of hMSCs in the GVHD target organs and to answer whether hMSCs home to these organs, hMSCs were stained with CM-Dil and injected into host mice at the time of transplantation. Migration patterns were observed towards the liver, colon, lungs and spleen. 6 days after transplantation, hMSCs can be localized in the liver, colon lung and spleen of GHVD host mice (Figure 1A-B)*.*

EphB2 and ephrin-B2 are reported to be expressed by MSCs [18]. Measuring their mRNA levels in host spleen, colon and lungs by RT-qPCR gave us a clue of possible hMSC migration to these organs. 6 days after transplantation, we noted increased levels of EphB2 and ephrin-B2 in host mice spleen, colon and lungs treated with hMSCs (Figure 1C) (Additional file 1).

Treatment with hMSCs protects GVHD mice from death, results to lower GVHD scores, decreases pathologic changes of GVHD in target organs and suppresses early donor T cell alloreactivity.

**Figure 2:**
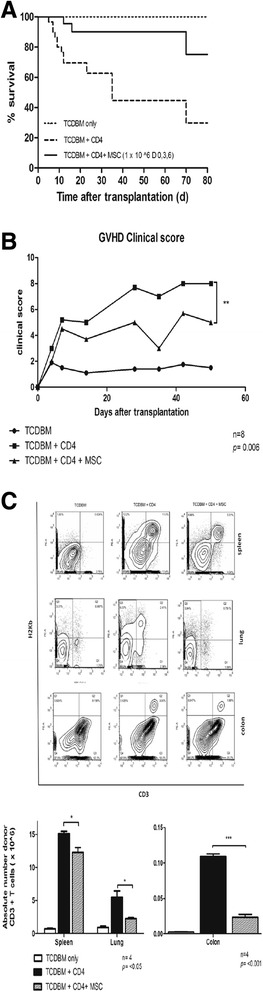
**Determination of hMSC effect in alloreactivity. (A)** Differences in survival, **(B)** GVHD clinical score, and **(C)** donor T cell expansion in the 3 groups of mice. Lethally irradiated BALB/c host mice were given intravenous injections of 2 × 10^6 TCDBM cells from C57BL/6 donors with or without 0.25 × 10 ^6 CD4+ T cells from donors and 1x 10 ^6 hMSCs. Multiple doses of hMSCs at the day of transplantation then 3d and 6d after transplantation were given for observation of survival and GVHD score. A single dose of hMSCs was given at the day of transplantation for testing of donor T cell expansion. **(A)** Survival of irradiated hosts given TCDBM cells alone (*n =* 10) or with 0.25 × 10^6 CD4+ T cells (*n = *20) or with 0.25 × 10^6 CD4+ T cells and 1 × 10^6 hMSCs (*n = *12) (*p =* 0.0035). **(B)** GVHD score of host mice and **(C)** relative and absolute numbers of donor CD3 T cells in BALB/c host tissue 6d after transplantation with TCDBM alone or with CD4+ T cells , or CD4+ T cells and hMSCs. (*p <0.05). Data were combined from a single experiment with 8 mice per group for the GVHD scoring and 4 mice per group in the experiment for determination of donor T cell engraftment.

**Figure 3:**
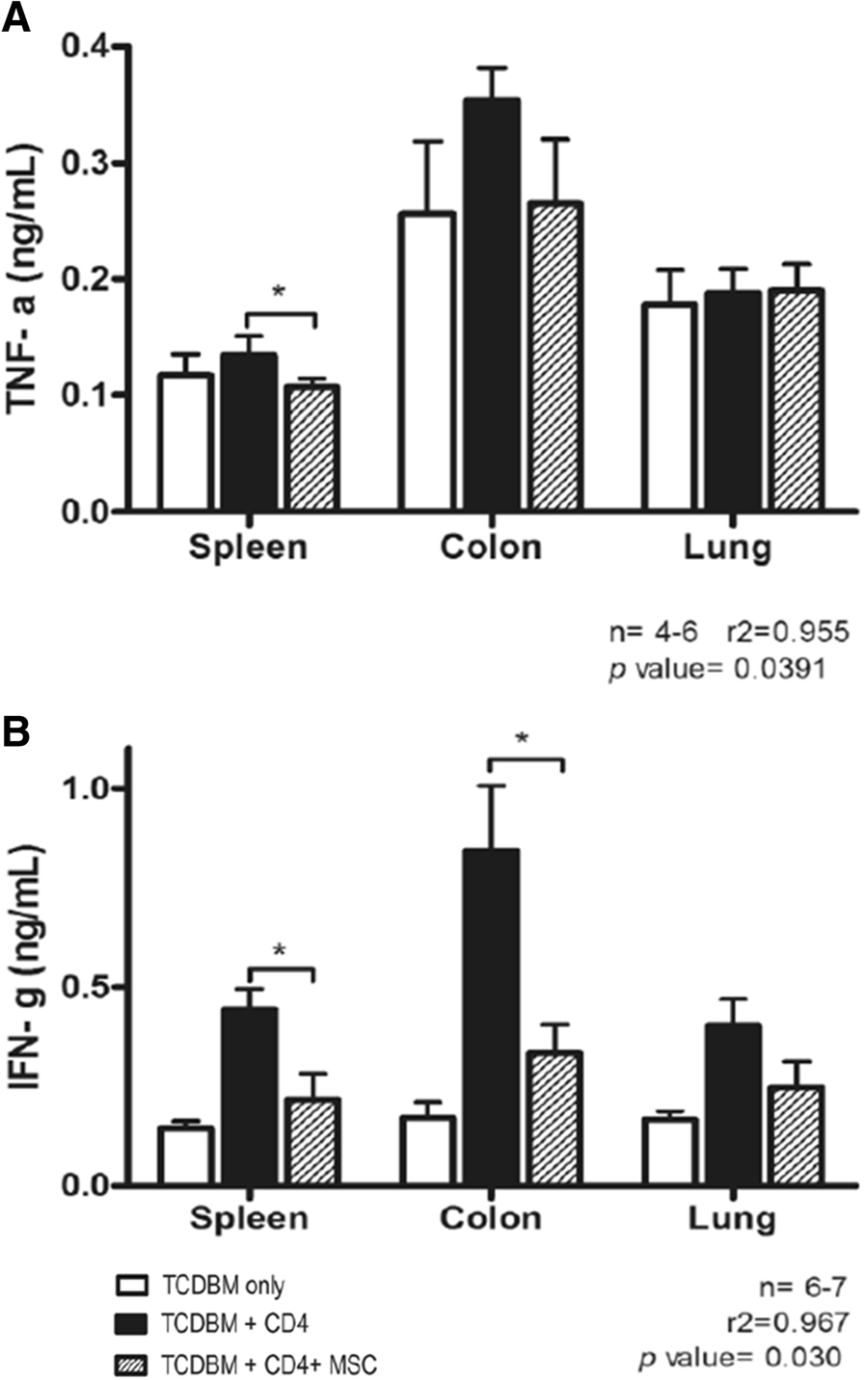
**Measurement of levels of proinflammatory cytokines. (A)** TNF-α. **(B)** IFN-γ. Lethally irradiated BALB/c host mice were given intravenous injections of 2 × 10^6 TCDBM cells from C57BL/6 donors with or without 0.25 × 10 ^6 CD4+ T cells from donors and 1 × 10^6 hMSCs. Tissue cytokine levels [TNF-α**(A)**, IFN- **(B)**] of BALB/c hosts 6d after transplantation *p<0.05. Data were combined from 2 independent experiments with 4 mice in each experiment.

**Figure 4:**
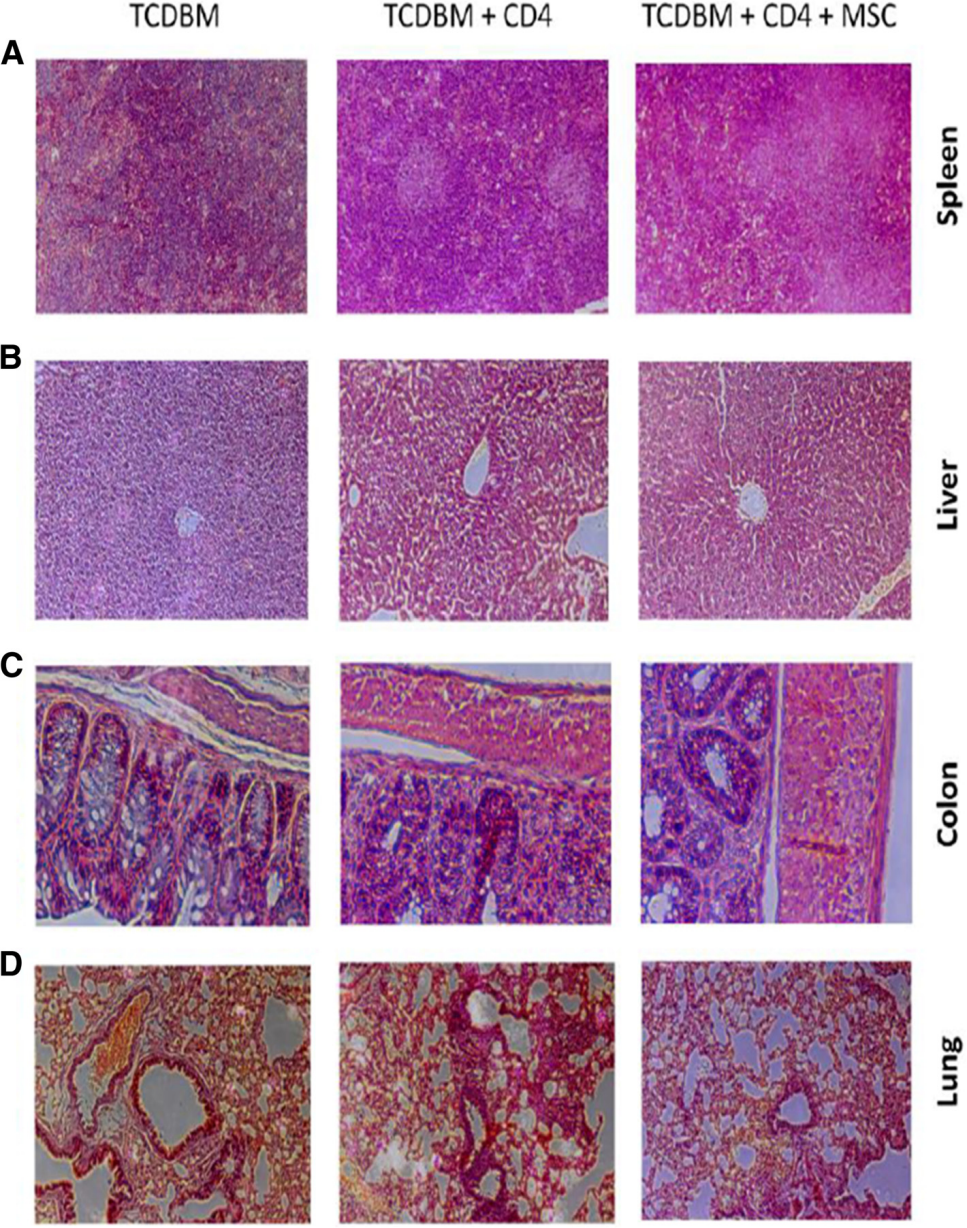
**Comparison of histopathological changes in host tissues of the 3 groups.** Lethally irradiated BALB/c host mice were given intravenous injections of 2 × 10^6 TCDBM cells from C57BL/6 donors with or without 0.25 × 10 ^6 CD4+ T cells from donors and 1 × 10 ^6 hMSCs. Multiple doses of hMSCs were given at the day of transplantation then 3d and 6d after transplantation. Host spleen, liver, colon and lung are harvested 14d after transplantation and hematoxylin and eosin sections were prepared for light microscopy. Diminished graft versus host response was noted upon treatment with hMSCs namely substantial white pulp cellular infiltration in the spleen, periportal inflammation in the liver, loss of crypts in the colon and peribronchial inflammation and alveolar congestion in the lungs. Each specimen is a representative of 3 mice from each treatment group. Magnification × 100 for panels **A** and **D**; × 200 for panels **B** and **C**.

Documentation of the host mice’s long term survival and GVHD score after being given multiple doses of hMSCs were performed (days 0, 3 and 6 after transplantation). Multiple doses are given to overcome the transient nature of the immunosuppressive effects of hMSCs and keep the mice alive for a longer period. All the negative control group host mice that received donor TCDBM cells survived for 80 days. The survival of positive control group host mice given TCDBM and CD4+ was approximately 30%, 80 days after transplantation. Maximal death rates were observed around days 7–14 after transplantation. The group of mice given hMSCs survived better than the positive control, with around 80% of mice still alive 80 days after transplantation. The differences in survival between the 3 groups are significant (*p =* 0.0035*)* by log-rank (Mantel-Cox Test) analysis of survival (Figure 2A). The clinical score also shows the effect hMSCs have in systemic symptoms of GVHD (*p* = 0.006) (Figure 2B). An important early indicator for acute GVHD is donor T cell expansion in the host mice. Treatment of mice with hMSCs caused a marked reduction in donor T cell alloreactivity in the target organs and lymphoid tissues (Figure 2C)*.* In line with these findings, a significant decrease in levels of proinflammatory cytokines in host tissue, notably TNF-α in the spleen (*p =* 0.0391*),* and IFN-γ in the spleen and colon was noted (*p =* 0.030) (Figure 3A-B)*.* Lastly, host spleen, liver, colon, and lungs were harvested 14 days after transplantation and were noted to have less prominent GVHD features with hMSC treatment (Figure 4).

### hMSCs cause increased production of iNOS

**Figure 5:**
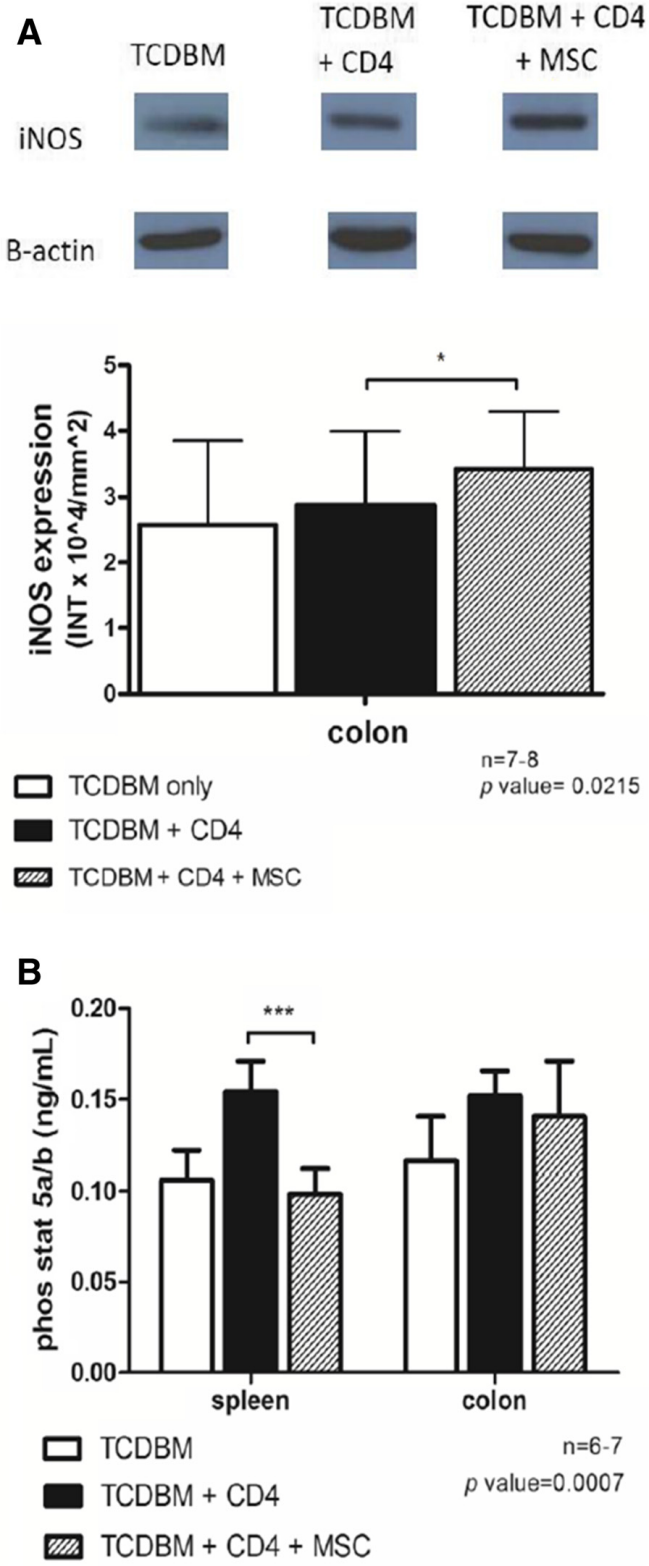
**Measurement of immunosuppressive mediators. (A)** iNOS production in host colon. **(B)** Phosphorylated STAT 5A/B levels in the host spleen and colon. Lethally irradiated BALB/c host mice were given intravenous injections of 2 × 10^6 TCDBM cells from C57BL/6 donors with or without 0.25 ×10 ^6 CD4+ T cells from donors and 1 × 10 ^6 hMSCs. **(A)** Host colon was harvested 6d after transplantation and iNOS expression detected by western blotting method. Each lane contains 50 ug of protein. B-actin served as loading control. Intensity of bands is expressed in INT × 10^4/mm^2 units and analyzed using The Discovery series Quantity 1D Analysis software, Biorad Laboratories, Inc., USA. *p = 0.0215. Data were combined from 3 independent experiments with 2-3 mice in each experiment. **(B)** For testing the levels of phosphorylated STAT 5A/B in the host spleen and colon, tissues were harvested 6d after transplantation and levels were determined by using ELISA. (***p = 0.0007). Data were combined from 2 independent experiments with 3-4 mice in each experiment.

An increase in the production of the immunosuppressive soluble mediator, nitric oxide, catalyzed by inducible nitric oxide synthase (iNOS), plays a role in the mechanism of immunosupression caused by MSCs, such as suppressing proteins regulating T cell cycle proliferation. A significant increase in levels of iNOS was noted in the host colon upon treatment with hMSCs (*p =* 0.0215*)* (Figure 5A).

### hMSCs cause suppression of STAT 5A/B phosphorylation

Suppression of signal transducer and activator of transcription 5A/B (STAT 5A/B) phosphorylation is a known key factor in T cell proliferation and may be a possible explanation for the reduction in the number of T cells in the target tissues ultimately resulting to suppression of graft versus host reaction (GVHR). This phenomenon was most notably observed in the host splenocytes where there is very high T cell activity (*p =* 0.0007*)* but was not significant in the colon (*p =* 0.7216*)* (Figure 5B)*.*

### hMSCs suppress expression of chemokine receptors CCR5 and CXCR3 and cognate ligands RANTES, CCL3 and CXCL9

**Figure 6:**
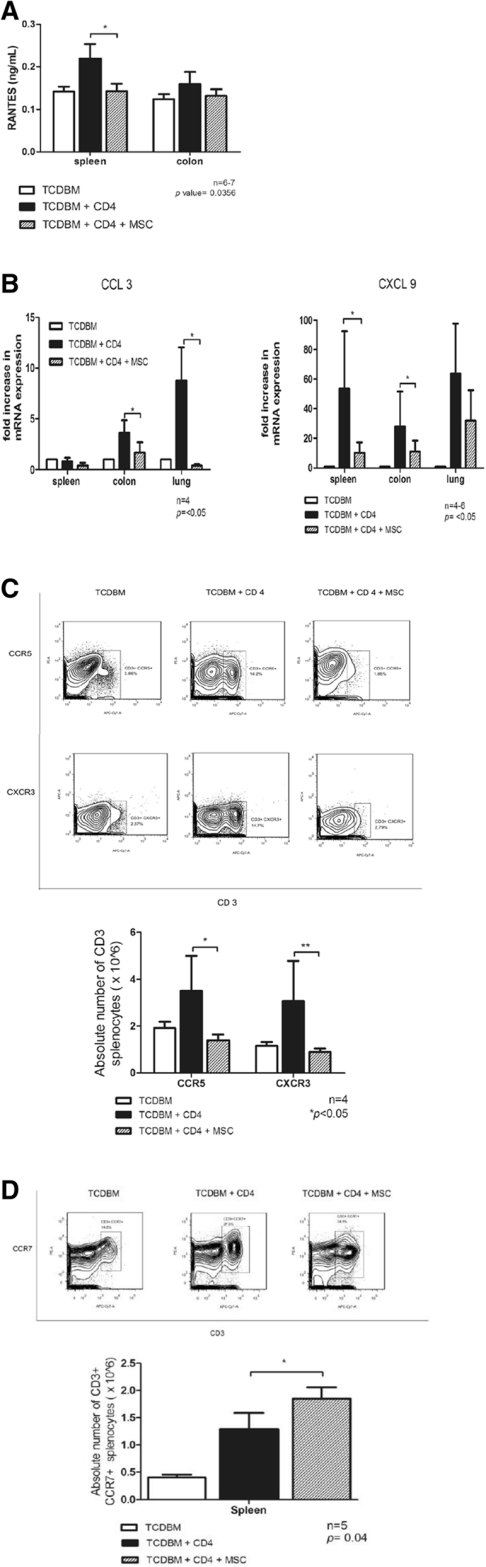
**Chemokine expression in host spleen and target organs. (A)** RANTES expression. **(B)** Expression of CCL3 and CXCL9.** (C-D)** Expression of CCR5, CXCR3 and CCR7. Lethally irradiated BALB/c host mice were given intravenous injections of 2 × 10^6 TCDBM cells from C57BL/6 donors with or without 0.25 × 10 ^6 CD4+ T cells from donors and 1 × 10 ^6 hMSCs. **(A)** Host spleen and colon were harvested 6d after transplantation and RANTES expression measured using ELISA (*p = 0.0356). Data were combined from 2 independent experiments with 3-4 mice in each experiment. **(B)** Expression of CCL3 and CXCL9 6d after transplantation in BALB/c host spleen, colon and lung were measured by RT-qPCR. Data were combined from 2 independent experiments with 4-6 mice. **(C,D)** Expression of CCR5, CXCR3 and CCR7 6d after transplantation in BALB/c host spleen were measured by using trypan blue cell exclusion assay and flow cytometry and expressed in absolute numbers of CD3+ CCR5+, CD3+ CXCR3+ **(C)** and CD3+ CCR7+ **(D)** splenocytes. Data were combined from 2 independent experiments with 2-4 mice in each experiment (*p<0.005).

Chemokine receptors expressed by donor T cells and chemokine ligands expressed by GVHD target tissues play a critical role in donor T cell alloreactivity in GVHD [19]. In terms of the ligands, a significant decrease in expression of RANTES (CCL5) in host splenocytes (*p =* 0.0356*)* but not in the colon (*p =* 0.3214) was noted (Figure 6A). Analysis via RT-qPCR of chemokine ligands CCL3 and CXCL9 was also performed. Suppression of these ligands in the host spleen, colon and lungs was noted with hMSC treatment (Figure 6B). Moreover, the number of cells expressing the corresponding receptors CCR5 (receptor for CCL3 and RANTES) and CXCR3 (receptor for CXCL9) are also significantly decreased in the spleen with hMSC treatment (Figure 6C).

### hMSCs increase the numbers of cells expressing the chemokine receptor CCR7

CCR7 prolonged survival of GVHD mice via increasing the trafficking of MSCs to lymphoid organs [20]. We noted that 6 days after transplantation, the number of cells expressing CD3+ CCR7+ cell population increased with hMSC treatment in the spleen of host mice (*p* = 0.04) (Figure 6D).

## Discussion

In this model, administration of hMSCs to nonhumanized GVHD mouse models was performed. Immunosuppression was noted despite the probable immune reactivity of human cells to murine cells. The hMSCs served as a niche for engraftment in hematopoietic stem cell transplantation in the murine models regardless of the MSC source. Demonstration of the presence of hMSCs in the host tissues was done via *in vivo* imaging. hMSCs were localized in the liver, colon, lung and spleen 6 days after transplantation. CM-Dil was used because it is known not to fade or leak to other cells even after a long period [21]. The measurement of gene expression of EphB2 and ephrin-B2, done during the same period, also enabled documentation of probable homing of hMSCs to lymphoid organs and target tissues. These members of the erythropoietin-producing hepatocellular (Eph) receptors and ligands were reported to be expressed by hMSCs and contribute to its immunosuppressive activities via downregulation of T-cell expression of soluble mediators such as IFN-γ, TNF-α, IL-2 and IL-17. Other members of this family are important regulators of angiogenesis and induction of inducible nitric oxide synthase (iNOS) expression, an important mediator of the immunosuppressive activity of MSCs. Moreover, binding of these receptors and ligands to cognate receptors and ligands in T lymphocytes was reported to decrease T lymphocyte proliferation [22]. Mice given hMSCs survived better as compared to positive control mice. Mice treated with hMSCs also had less severe symptoms of GVHD as evidenced by lower GVHD scores. Further investigation was done on the various mechanisms that may support this phenomenon. Pathologic evidence of resolution of GVHR in the spleen, lung, liver and colon was noted 14 days after transplantation. With active GVHR, the spleen usually manifests pathologically as prominent cellular infiltration in the splenic white pulp. In the lungs, this reaction is observed as inflammatory cells that can be localized at the region surrounding the bronchioles. Lung GVHR also presents as areas of congestion in the alveolar areas. These inflammatory cells of GVHR are seen surrounding the portal vein in the liver. The prominent feature of GVHR in the colon, apart from inflammatory cellular infiltration is colonic crypt drop outs [23-26]. With hMSC treatment these changes were noticeably abated. Suppression of expansion of donor cells in the spleen, lung and colon was noted 6 days after transplantation. This phenomenon most probably is not a result of T cell apoptosis [27]. This results to subsequent dampening of the Th1 response and the release of proinflammatory cytokines IFN-γ and TNF-α in the tissues [16,17,22,28]. Previous studies however demonstrated that the immunosuppressive activities of MSCs are dependent on IFN-γ [11,29,30]. This means that initially an environment with high levels of IFN-γ is necessary for hMSCs to exert their immunosuppressive effects and upon onset of activity of hMSCs in the lymphoid organs and target tissues (i.e. 6 days after transplantation) the levels of proinflammatory cytokines decrease. Chemokine ligands and receptors play a major role in alloreactivity and probably the immunomodulatory activities employed by hMSCs. RANTES (CCL-5) is produced by various cell types that are involved in the allogeneic response such as macrophages, lymphocytes, dendritic cells and endothelial cells and causes recruitment of other cells to migrate to sites of inflammation such as activated T cells, monocytes, macrophages, dendritic cells and neutrophils [31]. In addition to RANTES, CCL3 and CXCL9 are chemokine ligands expressed by target tissues of GVHD and cause migration of mainly Th1 cell subset population to the said tissues. These ligands bind to their cognate receptors CCR5 for CCL3 and RANTES, and CXCR3 for CXCL9 [19]. These ligands and receptors are reported to be upregulated in lymphoid tissues concomitant with the increase in levels of IFN-γ at around 3 days after transplantation [12]. This increased chemokine receptor-ligand expression are probably necessary for migration of MSCs to lymphoid organs before they home to target tissues to exert their immunosuppressive actions. We suspect that chemokine expression is initially upregulated for migration of hMSCs to lymphoid organs, and later on (6 days after transplantation) suppressed by the time enough hMSCs are in the area to exert immunosuppressive actions. This way the hMSCs are already in the lymphoid organs and target tissues ready to release soluble immunosuppressive mediators, at the same time, the downregulation of chemokine receptors and ligands limits migration of inflammatory cells to these areas all in all resulting to reduction of GVHR. With CCR7, the response may be somehow different. CCR7 is a G-protein coupled receptor expressed on T cells. They function to direct T-cells to the lymphoid organs [32]. In a recent study, MSCs transfected with CCR7 prolonged survival of GVHD mice via directing migration of these CCR7 transfected MSCs to secondary lymphoid organs. This increased migration of MSCs causes immunosppression of donor T cells in the vicinity of the MSCs [20]. The observation of increased number of CD3+ CCR7+ cell population with hMSC treatment in the spleen of host mice probably promotes the migration of hMSCs to lymphoid organs and target organs for more efficient immunosuppression.

Inducible nitric oxide synthase (iNOS) is an enzyme that catalyzes the production of nitrous oxide which has been reported to affect T cell receptor signaling, cytokine receptor expression and production [12]. Produced by MSCs, they are also reported to cause suppression of STAT5 phosphorylation, causing probable inhibition of T cell proliferation [33]. STAT 5 is part of the Jak/STAT signaling pathway which is activated by tyrosine kinases. Phosphorylation of this protein results to their migration to the nucleus where they function as transcription factors regulating T cell proliferation [34]. An increase in iNOS production by hMSCs, accompanied by a simultaneous suppression of STAT5A/B phosphorylation thereby causing decreased T cell proliferation was noted. These activities cumulatively results to immunosuppression and may explain the improvement of GVHD scores and survival rates of hMSC treated host GVHD mice.

To date there are conflicting reports whether cell to cell contact between MSCs and T cells or soluble factors are the main mediators of T cell suppression [29,35]. The recent reports of interaction between members of the ephrin family of receptors and ligands in both MSCs and T cells and wide studies on soluble factors reported to be secreted by MSCs, such as IDO, NO, PGE2 and HLA-G5, most likely implies that the immunosuppressive mechanism elicited by MSCs may be a combination of the two [22,36]. GVL experiments were not performed using this model. Previous reports demonstrated that MSCs may not contribute to GVL but on the contrary may enhance tumor metastasis through secretion of specific soluble factors [37]. Another study reported that the immunosuppressive effect of MSCs favors tumor growth in allogeneic animals [38].

## Conclusions

**Figure 7:**
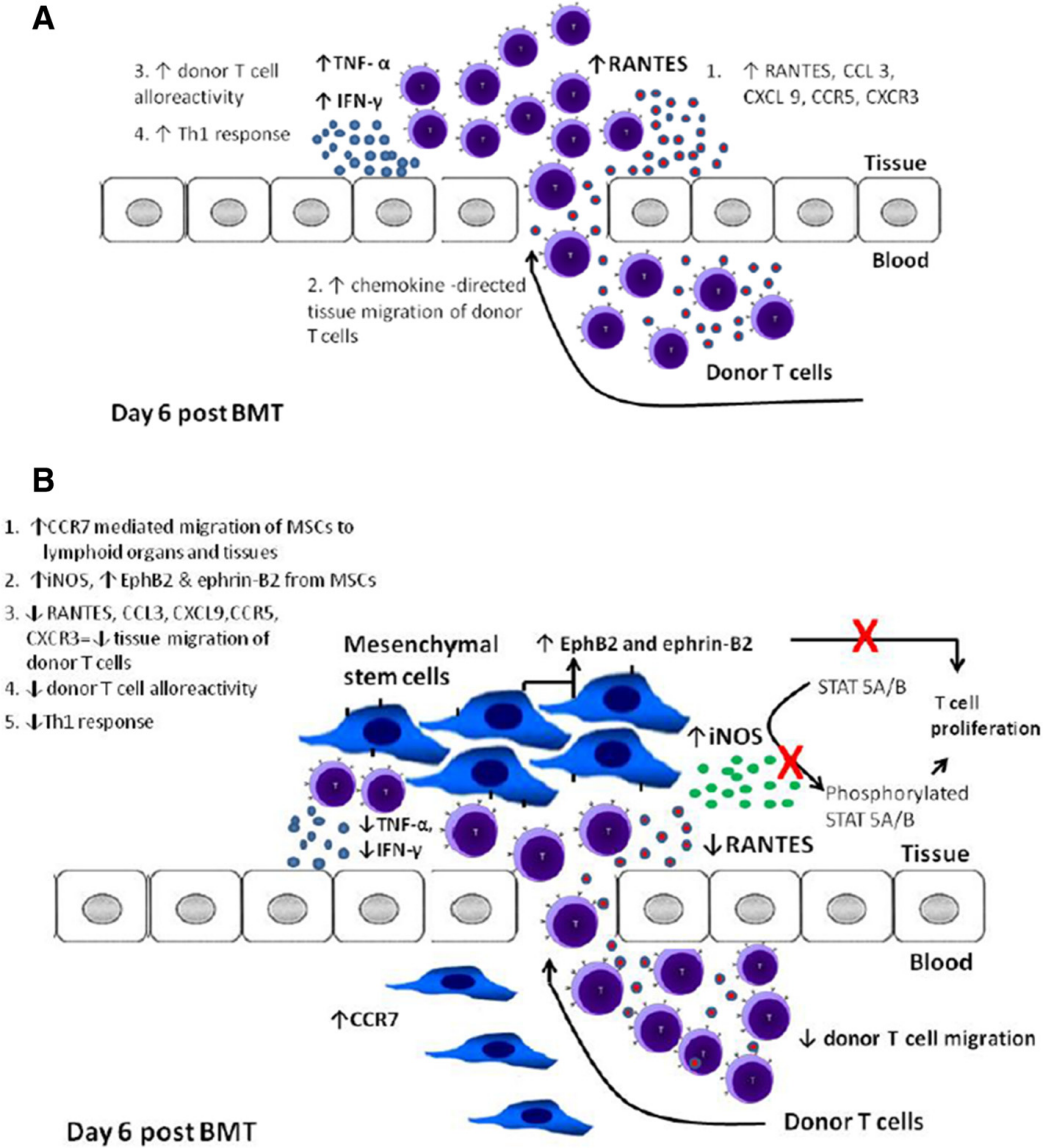
**Immunosuppressive mechanisms of hMSCS in GVHD mouse models. (A)** In the absence of hMSCs, upregulation of chemokine receptors and ligands causes migration of donor T cells to the target tissues of GVHD causing increased T cell alloreactivity and Th1 response. **(B)** With hMSC treatment, CCR7 increases migration of hMSCs to lymphoid organs and target tissues, then hMSCs produce soluble mediators (iNOS) causing immunosuppression. These soluble mediators suppress ligands CCL3, RANTES and CXCL9 and corresponding receptors CCR5 and CXCR3 with resultant decresased migration of donor T cells to the target tissues. This subsequently results to decreased TNF-α and IFN- γ. iNOS also suppresses phosphorylation of STAT 5A/B proteins that regulates T cell proliferation. EphB2 and ephrin-B2 which are specifically found in hMSCs also binds with T cells and suppress their proliferation.

The mechanisms of immunosuppression brought about by MSC treatment in GVHD remains elusive. hMSCs infused to GVHD murine models increase the number of cells expressing CCR7 contributing to homing of hMSCs to lymphoid organs and then to target tissues of GVHD. hMSCs release iNOS to suppress phosphorylation of STAT 5A/B proteins and control T cell replication. The chemokine ligands RANTES, CCL3 and CXCL9 are suppressed along with their corresponding receptors CCR5 and CXCR3 resulting to diminished migration of alloreactive donor T cells to lymphoid organs and target tissues and cytokine response. (Figure 7) Ultimately, these mechanisms result to decreased GVHR which is reflective in the GVHD scores, histopathologic analysis of GVHD target tissues and survival rates.

## Methods

### Animals

Wild-type C57BL/6 (H-2Kb) and BALB/c (H-2Kd) male mice 6 to 8 weeks old were purchased from the breeding facility of the Laboratory Animal Unit of the University of Hong Kong. All mice were kept in a specific pathogen-free facility. Animals were kept based on the guidelines set by the Committee on the Use of Live Animals in Teaching and Research (CULATR) of the University of Hong Kong and the Department of Health Hong Kong.

### hMSC culture and preparation of cell suspensions

hMSCs were obtained from bone marrow of three healthy adult donors with the approval of the ethics committee institutional review board of the Queen Mary Hospital, The University of Hong Kong, cultured and propagated using low glucose, L-glutamine supplemented Dulbecco’s Modified Eagle Medium, 100 U/mL penicillin and 100 mg/mL streptomycin (Life Technologies, Carlsbad, CA). Prior to cell passaging every 3-4 days, cells were lifted using 0.05% Trypsin solution (Thermo Fisher Scientific, Waltham, MA) with gentle scraping using a 300 mm cell scraper (TPP, Trasadingen, Switzerland). Cells were used at 5th-30th passage.

Single cell suspensions were prepared from donor mice spleens, washed twice and filtered through a fine metallic mesh. Mononuclear cells were isolated from the solution using Lymphoprep Density Gradient Medium. (Stemcell Technologies, Vancouver, BC). The isolate was then enriched for CD4+ cells with anti-mouse CD4 magnetic microbeads using the MidiMACS system (Miltenyi Biotech, Auburn, CA). The enriched cells were stained with FITC anti-mouse CD4 (eBioscience, San Diego, CA) and purity was ascertained at 95% per cent or more. TCDBM cells were prepared from donor mice femurs, washed and filtered using a metallic mesh, stained with anti-mouse CD90.2 (Thy1.2) Biotin (eBioscience, San Diego, CA) and streptavidin microbeads (Miltenyi Biotec, Auburn, CA) and passed over two consecutive MACS LS separation columns. Adequacy of depletion was checked by staining the eluate with APC/Cy7 anti-mouse CD3 (Biolegend, San Diego, CA) and ascertaining that the percentage of CD3+ cells is less than 5%.

### Construction of GVHD model and clinical scoring

Induction of GVHD was done by lethal irradiation of BALB/c host mice (800 cGy dose) using Gamma 3000 Elan as radiation source (Best Theatronics, Ottawa, Canada). Donor cells and hMSCs were prepared and cultured as mentioned above and injected via tail vein within 24 hours. Host mice received either 2 × 10^6 TCDBM cells (negative control), 2 × 10 ^6 TCDBM cells and 0.25 × 10^6 CD4+ spleen cells (positive control) or 2 × 10^6 TCDBM cells, 0.25 × 10^6 CD4+ spleen cells and 1 × 10^6 hMSCs (treatment group). In all of the experiments except for histopathologic analysis, analysis of survival and GVHD score, 1 × 10^6 hMSCs are given at the day of transplantation. For these three experiments, 1 × 10^6 hMSCs were given at the day of transplantation, then at days 3 and 6 after transplantation. They were given autoclaved water and feeds during the first 21 days after transplantation. The presence of GVHD was assessed every day based on weight loss, posture, activity, fur texture and skin integrity based on an established criteria [39]. Survival and the presence of diarrhea were also recorded every day. In compliance with the university’s committee on the use of live animals in teaching and research, mice were euthanized for weight loss of >20% from its baseline weight.

### Histopathologic analysis of GVHD

Histopathologic specimens from the host spleen, colon, liver and lungs were harvested 14 days after transplantation and fixed in 10% formaldehyde and embedded into paraffin blocks. Sections of 4–5 um thickness were obtained and stained with hematoxylin and eosin and viewed under a light microscope for cellular and tissue changes of acute GVHD (Carl Zeiss Axiovert 40 CFL, Oberkochen, Germany). Images are captured using Canon EOS 600D (Chichibu, STM, Japan) and are exported as a series of .jpg files.

### Detection of donor cell engraftment and chemokine receptor expression

Single cell suspensions were prepared from host spleen, lung, and colon by mechanical disruption and filtering using a metallic mesh. Live cells were quantified by using Trypan blue exclusion cell assay. The following fluorescent-conjugated antibodies were used for flow cytometry: APC/Cy7 anti-mouse CD3 (Biolegend, San Diego, CA), PE anti-mouse H-2K^b, APC anti-mouse CXCR3, PE anti-mouse CCR5, PE anti-mouse CCR7 (eBioscience, San Diego, CA). Samples were run using a four fixed-aligned laser flow cytometer and data were analyzed using BD FACS Diva^TM software (Becton Dickinson, Mountain View, CA) and FlowJo software (TreeStar, Ashland, OR).

### Detection of TNF-α, IFN-γ, RANTES, phosphorylated STAT 5A/B and inducible nitric oxide synthase

Specimens from host spleen, lung and colon were mechanically disrupted and homogenized using a handheld homogenizer (Omni International, Kennesaw, GA). Supernatants were collected for ELISA of tumor necrosis alpha (TNF-α), interferon gamma (IFN-γ), RANTES/CCL-5 and phosphorylated STAT 5A/B (eBioscience, San Diego, CA). Inducible nitric oxide synthase (iNOS) levels were detected using polyclonal antibodies (Abcam, Cambridge, UK) for western blotting. Total protein concentration in the supernantant was measured using Bradford’s reagent. Supernatants were then subjected to sodium dodecyl sulfate-polyacrylamide gel electrophoresis (SDS-PAGE). Proteins were then transferred from the gel to nitrocellulose membranes and ECL reagent was used for visualization of the protein (GE Healthcare, Buckinghamshire, UK).

### Quantification of chemokine ligand and EphB2 and ephrin-B2 expression by real time RT-qPCR

Frozen BALB/c host spleen, lung and colon tissues harvested 6 days after transplantation were mechanically disrupted and homogenized as previously described and total tissue RNA was isolated using a RNA extraction kit. The isolated RNA is then subjected to reverse transcription using oligo dT primer and PrimeScript reverse transcript enzyme according to protocol provided by the manufacturers. The synthesized cDNA was used for quantification of mRNA by real-time quantitative PCR by using SYBR premix Ex Taq II (Tli RNaseH Plus) and 7900HT Fast Real-Time PCR System (Applied Biosystems, Foster City, CA, USA) (All kits and reagents from Takara Bio Inc., Otsu-shi, Japan). The forward and reverse sequence for CCL3, CXCL9, EphB2, ephrin-B2 were obtained from previously published data [40,41]. Gene expression was calculated using the 2^-ΔCT method relative to GADPH (glyceraldehyde 3-phosphate dehydrogenase) CT values and were presented relative to the expression of recipients given TCDBM only.

### Localization of hMSCs in target tissues

hMSCs were stained with CM-Dil cell labeling solution for 20 minutes at 37°C and washed 3 times (Life Technologies, Carlsbad, CA). 1 × 10^6 cells were given via tail vein to host mice and at 6 days post transplantation, spleen, liver, lung and colon were harvested and fluorescent signal intensity of the stained cells in the tissues were measured at 550 nm Abs spectrum using an in vivo imaging platform for small animals. (CRi Maestro II, Cambridge Research & Instrumentation, Inc., Woburn, MA).

### Statistical analysis

Analysis of mice survival was done by constructing Kaplan-Meier survival curves using Prism Version 5.01 (GraphPad Software, San Diego, CA). Statistical differences in animal survival were analyzed by log-rank (Mantel-Cox) test. Differences in fluorescence signals using *in vivo* imaging were analyzed using Student t test to compare the group treated with hMSC with the group not treated with hMSC. Differences in GVHD score, recovery of donor cells in host tissue, chemokine expression, EphB2 and ephrin-B2 expression, inflammatory cytokine levels, inducible nitric oxide synthase production, phosphorylated T-cell cycle regulatory protein levels, were analyzed using Student t test to compare the positive control and treatment groups. For all tests, a *p* value of .05 or less was considered significant.

## Additional file

Additional file 1: Table S1. Primer Sequences.

## Abbreviations

cDNA: Core deoxyribonucleic acid
CM-Dil: 1,1′-dioctadecyl-3,3,3,3′-tetramethylindocarbocyanine perchlorate
DNA: Deoxyribonucleic acid
ELISA: Enzyme linked immunosorbent assay
GADPH: Glyceraldehyde 3-phosphate dehydrogenase
GVHD: Graft versus host disease
GVHR: Graft versus host reaction
GVL: Graft versus leukemia
HLA: Human leukocyte antigen
hMSC: Human mesenchymal stromal cells
IDO: Indoleamine-2,3-dioxygenase
IFN-γ: Interferon-gamma
IL: Interleukin
iNOS: Inducible nitric oxide synthase
mRNA: Messenger ribonucleic acid
MSC: Mesenchymal stromal cells
PCR: Polymerase chain reaction
PGE: Prostaglandin E2
RANTES: Regulated on activation normal T cell expressed and secreted
RNA: Ribonucleic acid
RTqPCR: Reverse transcriptase quantitative polymerase chain reaction
SDS- PAGE: Sodium dodecyl sulfate polyacrylamide gel electrophoresis
STAT 5: Signal Transducer and Activator of Transcription 5
TCDBM: T cell depleted bone marrow
TNF-α: Tumor necrosis factor- alpha
