# Supplementary figures and images for: Immunosuppressive mechanisms of human bone marrow derived mesenchymal stromal cells in BALB/c host graft versus host disease murine models
Source: Exp Hematol Oncol. 2015 Apr 30;4:13. doi: 10.1186/s40164-015-0007-0 (PMC4440561; doi:10.1186/s40164-015-0007-0)

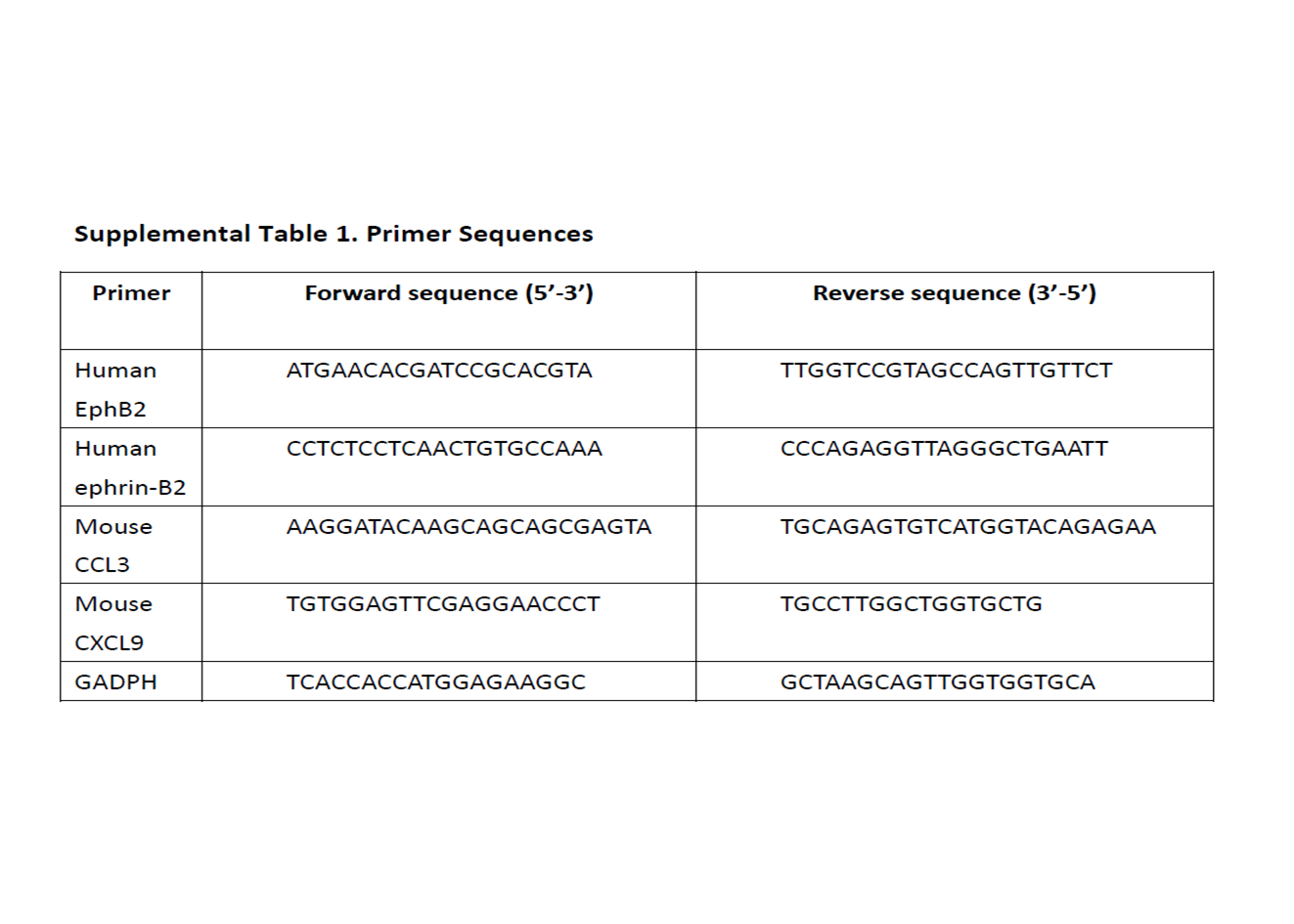

Supplement: Additional file 1: Table S1. — Primer Sequences. [file 40164_2015_7_MOESM1_ESM.tiff]
